# *Lactobacillus plantarum* ST-III culture supernatant protects against acute alcohol-induced liver and intestinal injury

**DOI:** 10.18632/aging.205331

**Published:** 2023-12-19

**Authors:** Feng Xu, Zengqiang Chen, Longteng Xie, Shizhuo Yang, Yuying Li, Junnan Wu, Yuyu Wu, Siyuan Li, Xie Zhang, Yanyan Ma, Yanlong Liu, Aibing Zeng, Zeping Xu

**Affiliations:** 1 Department of Gastroenterology, Ningbo Medical Center Li Huili Hospital, The Affiliated Hospital of Ningbo University, Ningbo 315000, China; 2 Healthcare Center, The First Affiliated Hospital of Wenzhou Medical University, Wenzhou 325000, China; 3 Department of Infection Diseases, The Affiliated Xiangshan Hospital of Wenzhou Medical University, Ningbo 315700, China; 4 School of Pharmaceutical Sciences, Wenzhou Medical University, Wenzhou 325035, China; 5 Ruian People's Hospital, Wenzhou Medical College Affiliated Third Hospital, Wenzhou 325200, China; 6 School of Mental Health, Wenzhou Medical University, Wenzhou 325035, China; 7 Department of Pharmacy, Ningbo Medical Center Li Huili Hospital, The Affiliated Hospital of Ningbo University, Ningbo 315000, China; 8 School of Laboratory Medicine and Life Sciences, Wenzhou Medical University, Wenzhou 325035, China

**Keywords:** lactobacillus plantarum ST-III, alcoholic liver disease, oxidative stress, ER stress, inflammatory

## Abstract

The beneficial effects of probiotics have been studied in inflammatory bowel disease, nonalcoholic steatohepatitis, and alcoholic liver disease (ALD). Probiotic supplements are safer and more effective; however, their potential mechanisms are unclear. An objective of the current study was to examine the effects of extracellular products of *Lactobacillus plantarum* on acute alcoholic liver injury. Mice on a standard chow diet were supplemented with *Lactobacillus plantarum* ST-III culture supernatant (LP-cs) for two weeks and administered alcohol at 6 g/kg body weight by gavage. Alcohol-induced liver injury was assessed by measuring plasma alanine aminotransferase activity levels and triglyceride content determined liver steatosis. Intestinal damage and tight junctions were assessed using histochemical staining. LP-cs significantly inhibited alcohol-induced fat accumulation, inflammation, and apoptosis by inhibiting oxidative stress and endoplasmic reticulum stress. LP-cs significantly inhibited alcohol-induced intestinal injury and endotoxemia. These findings suggest that LP-cs alleviates acute alcohol-induced liver damage by inhibiting oxidative stress and endoplasmic reticulum stress via one mechanism and suppressing alcohol-induced increased intestinal permeability and endotoxemia via another mechanism. LP-cs supplements are a novel strategy for ALD prevention and treatment.

## INTRODUCTION

Alcohol-related liver disease (ALD) causes approximately 25 % of deaths associated with alcohol consumption [1, 2]. ALD includes parenchymal injury, fibrosis, inflammation, cirrhosis, and hepatocellular carcinoma [3]. Ethanol directly damages the liver by increasing oxidative stress, endoplasmic reticulum (ER) stress, inflammatory responses, and apoptosis [4]. Oxidative stress is activated during alcohol metabolism, which produces excessive reactive oxygen species (ROS) in hepatocytes [5]. ROS activates lipid synthesis genes to protect liver cells from alcohol toxicity and causes the accumulation of lipids in liver cells, finally developing into other alcoholic liver diseases [6, 7]. Alcohol damages intestinal epithelial barrier function and promotes intestinal permeability, endotoxin leakage, and serum endotoxin levels, leading to alcoholic liver damage [1]. The liver-gut theory of alcoholic fatty liver disease states that alcohol exposure increases intestinal permeability, releases gram-negative bacterial lipopolysaccharide (LPS), and activates immune responses [2]. LPS enters the liver through the enterohepatic axis and activates Kupffer cells to release inflammatory interleukins (e.g., IL-1β and IL-6) and tumor necrosis factor-alpha (TNF-α), resulting in ALD [8].

Despite extensive studies of ALD pathophysiology, there are no targeted therapies available. The treatment approach for ALD remains corticosteroids (or pentoxifylline as an alternative if steroids are contraindicated), abstinence, and nutritional support [9]. In recent years, researchers have turned their attention to probiotics, which possess many beneficial effects on intestinal function, including promoting intestinal development and mucosal immunity, reducing intestinal oxidative stress, alleviating diarrhea, and improving or maintaining gut barrier function [10]. They positively regulate intestinal flora, temporarily colonize the gastrointestinal tract, correct gut dysbiosis, and inhibit the growth and virulence of several enteric pathogens [8]. The therapeutic potential of probiotics was reported in animal models of ALD and clinical studies [11–13]. Human studies highlighted that the administration of probiotics improved liver function by decreasing oxidative damage/stress [14]. A clinical study showed that supplementation with *Lactobacillus plantarum* 8PA3 and *Bifidobacterium bifidum* restored intestinal flora and improved liver enzyme levels in patients with ALD [15]. *Lactobacillus rhamnosus* CCFM1107 protects against alcoholic liver injury (ALI) by restoring bowel flora and reducing oxidative stress [16].

Several studies showed that probiotics protected the liver from alcohol injury; however, no consensus intervention exists. Another study showed that *Lactobacillus rhamnoses* supernatant maintained the integrity of the intestinal barrier and attenuated endotoxemia-centered liver injury [17]. Heat-killed lactic acid bacteria cells also reduced inflammation and oxidative stress in ALI [18]. These studies above reported *Lactobacillus*, supernatant of *Lactobacillus*, and heat-killed *Lactobacillus* might mediate its benefits. However, some reports noted adverse cases with probiotic utilization, including fungemia [19], bacteremia [20], and worsened severe pancreatitis, with a high incidence of intestinal ischemia and mortality [21]. Furthermore, research on heat-killed bacteria is insufficient. These studies suggested that the supernatant might be safer and more effective.

*Lactobacillus plantarum* ST-III, isolated initially from Kimchi, Japan, underwent complete genome sequencing with analysis of the oligosaccharide metabolic pathway in 2011 [22]. Other studies showed that *Lactobacillus plantarum* ST-III was a potential probiotic for treating hyperlipidemia [23]. However, studies of *Lactobacillus plantarum* ST-III in alcoholic diseases have not been reported. The present study investigated LP-cs' effects on acute ALI and provided a potential ALD prevention and treatment strategy.

## MATERIALS AND METHODS

### Preparation of LP-cs

*Lactobacillus plantarum* ST-III AB161(CGMCC 22782) was provided by the Biological Experimental Center of Wenzhou Medical University. According to the instructions, LP was activated and passaged three times in MRS medium, inoculated in 100 mL liquid medium according to 3% inoculation amount of culture medium, cultured at 37° C, 5% CO_2_ for 24 hours, centrifuged 10 min at 4° C and 5000 r/min, and the supernatant was filtered through 0.22-μm filter to obtain extracellular fluid. This procedure was performed as described previously [17, 24].

### Animal studies

Male ICR mice (about 20 g) were obtained from Vital River Laboratory Animal Technology Co., Ltd, Shanghai. All mice were maintained and fed as described previously [17, 24]. LP-cs was added to the drinking water at 1:20, and 8–9 ml/mouse was consumed daily. During the two weeks of the experiment, the mice were maintained on the LP-cs treatment. Animals were given a 6 g/kg dose of ethanol via gavage and maintained fasting overnight with free access to drinking water containing LP-cs. All animals were anesthetized after 6 h of the experiment, and blood and tissue samples were collected for assays as described previously [17].

### Biochemical analysis

Plasma was collected by centrifuging the blood samples at 2,000 *g* for 30 min at 4° C. Plasma alanine aminotransferase (ALT) was tested using analytical reagent kits (C009-2-1, Nanjing Jiancheng Bioengineering Institute, China). Plasma LPS level was determined using enzyme-linked immunosorbent assay kits (50-658U, Lonza, Walkersville, MD). Liver tissues were homogenized and centrifuged according to the manufacturer's protocols. Then the levels of malondialdehyde (MDA), superoxide dismutase (SOD), and GSH-Px in the liver were determined using commercial kits, according to the manufacturer’s instructions (A003-1-2, A001-3-2, A005-1-2, Nanjing Jiancheng Bioengineering Institute, China).

### Hepatic triglyceride assay

Liver triglyceride levels were measured as described previously [25] (A110-1-1, Nanjing Jiancheng Bioengineering Institute, China).

### Liver TNF-α and IL-6 assays

Liver TNF-α and IL-6 were measured using TNF-α and IL-6 assay kits (900-K54, 88-7064-88, Thermo Scientific, Waltham, MA, USA) according to the manufacturer’s instructions.

### Western blot analysis

Liver tissues were homogenized, and western blotting was performed as previously described [26]. Membranes were probed using antibodies against cleaved caspase-3 (ab214430, Abcam) and GAPDH (# 2118S, Cell Signaling Technology). The protein signals were visualized by ChemiDocXRS+Imaging System (Bio-Rad, Hercules, CA, USA) and analyzed.

### Histological analysis, immunohistochemistry, and immunofluorescence

For hematoxylin and eosin (H&E) staining, paraformalin-fixed paraffin tissue sections were used to evaluate the characteristics of liver and intestinal tissues regarding histological changes and fibrosis. The immunohistochemistry procedure was performed as described previously [27], including the following primary antibodies CHOP, GRP78, PDI, and XBP-1 (sc-7351, sc-166490, sc-74551, and sc-8015, Santa Cruz Biotech, USA). The positive areas of CHOP, GRP78, PDI, and XBP-1 were recorded. For immunofluorescence, the tissue sections were blocked with 10% normal donkey serum for 1 h at 25° C in PBS and then incubated overnight with primary antibodies against claudin-1, occludin, and ZO-1 (sc-166338, sc-133256, sc-33725, Santa Cruz Biotech, USA) and P-gp (ab261736, Abcam) at 4° C. The nuclei were stained with Hoechst 33258 (0.25 l g/mL) dye. All fluorescence images were captured on a Nikon ECLIPSE Ti microscope.

### Terminal deoxynucleotidyl transferase deoxyuridine triphosphate nick-end labeling (TUNEL) assay

Four-millimeter liver sections were TUNEL stained according to the manufacturer's instructions using an ApopTag Peroxidase *In Situ* Apoptosis Detection Kit. The experiment methods above were performed as described previously [28].

### Quantitative real-time RT-PCR

The mRNA levels were determined using real-time PCR. Total RNA was isolated with TRIzol and reverse-transcribed according to the manufacturer’s protocol. The sequences of forward and reverse primers are listed in Table 1. The procedure was performed as described previously [17].

**Table 1:** Primer sequences for real-time RT-PCR.

**Gene**	**Source**	**Sequences (Forward/Reverse 5’-3’)**	
ACC	Mouse	GGGACTTCATGAATTTGCTGATTCTCAGTT	GTCATTACCATCTTCATTACCTCAATCTC
β-actin	Mouse	GGCTGTATTCCCCTCCATCG	CCAGTTGGTAACAATGCCATGT
FAS	Mouse	TGGGTTCTAGCCAGCAGAGT	ACCACCAGAGACCGTTATGC
NF-KB(P65)	Mouse	CTTGGCAACAGCACAGACC	GAGAAGTCCATGTCCGCAAT
PGC-1α	Mouse	AGACAAATGTGCTTCCAAAAAGAA	GAAGAGATAAAGTTGGTTTGGC
PPAR-α	Mouse	AGAGCCCCATCTGTCCTCTC	ACTGGTAGTCTGCAAAACCAAA
SREBP-1c	Mouse	GCGGAGCCATGGATTGCA	CTCTTCCTTGATACCAGGCCC

### Statistical analysis

All experiment data were expressed as the mean ± SEM and analyzed by one-way analysis of variance. Statistical differences were calculated using GraphPad Prism 8 (GraphPad Software, Inc., San Diego, CA, USA). Statistics are significant at *p*-values < 0.05.

## RESULTS

### LP-cs ameliorates acute alcohol-induced liver steatosis

**Figure 1:**
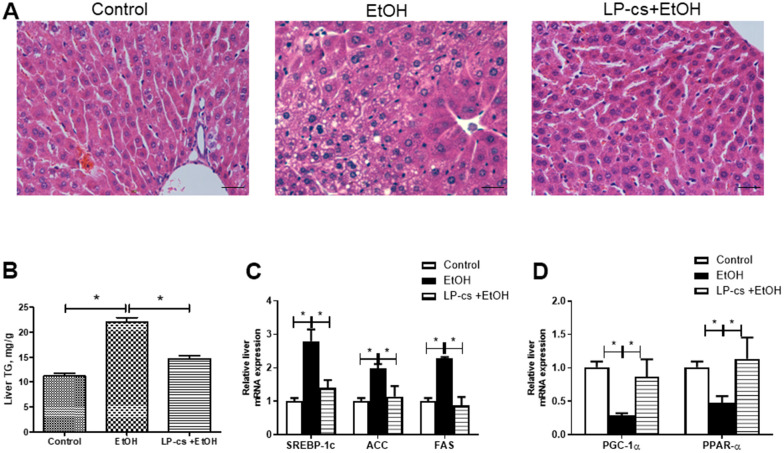
***Lactobacillus plantarum* ST-III culture supernatant (LP-cs) ameliorates acute alcohol-induced liver steatosis.** (**A**) Hematoxylin and eosin (H&E) staining of livers from Control, EtOH, and LP-cs +EtOH mice (40 x: scale bars = 25 μm). (**B**) Liver triglyceride (TG) levels. (**C**) Relative liver mRNA expression of SREBP-1c, acetyl-CoA carboxylase, and fatty acid synthase. (**D**) Relative liver mRNA expression of PGC-1α and PPAR-α. Data are expressed as mean ± SEM. *p < 0.05.

Biochemical and histological measurements were performed to investigate the effects of LP-cs on acute alcohol-induced liver steatosis. As expected, mice exposed to alcohol exhibited apparent hepatic lipid accumulation compared with control mice, while LP-cs pretreatment significantly prevented fatty liver induced by acute alcohol treatment (Figure 1A). As previously reported, liver triglyceride levels in the alcohol-exposed group were significantly higher than in the control group [17]. LP-cs pretreatment significantly decreased the acute alcohol-induced accumulated liver triglycerides (Figure 1B). Consistent with enhanced liver steatosis, the alcohol-exposed group showed increased expression of hepatic lipogenesis and decreased expression of hepatic fatty acid β-oxidation. Expression of the transcription factor SREBP1c in the alcohol group increased in the liver; it plays a pivotal role in lipogenic gene expression control. Accordingly, alcohol exposure enhanced expression of SREBP1c targets and fatty acid synthase, acetyl-CoA carboxylase (Figure 1C). Binge alcohol decreased gene transcript levels during fatty acid β-oxidation, including PGC-1α and PPAR-α. Pretreatment with LP-cs markedly reversed the changes of these genes induced by alcohol exposure (Figure 1C, 1D). These findings suggest that LP-cs pretreatment is essential in hepatic protection against alcohol-induced liver steatosis.

### LP-cs ameliorates acute alcohol-induced liver injury

**Figure 2:**
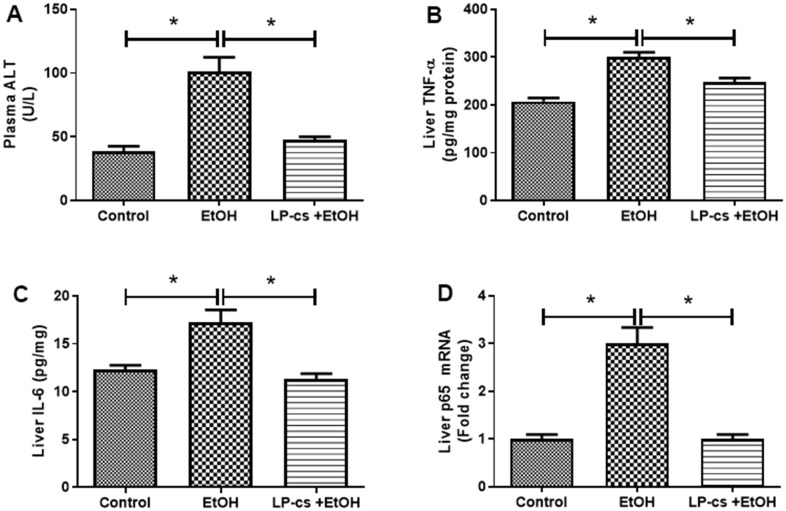
***Lactobacillus plantarum* ST-III culture supernatant (LP-cs) ameliorates acute alcohol-induced liver injury.** (**A**) Plasma alanine aminotransaminase (ALT) levels. (**B**) Liver TNF-α levels. (**C**) Liver IL-6 levels. (**D**) Relative liver mRNA expression of P65. Data are expressed as mean ± SEM. *p < 0.05.

Liver plasma was collected 6 h after binge alcohol administration. Plasma ALT levels were significantly elevated, and LP-cs pretreatment blocked this elevation at 6 h after alcohol treatment (Figure 2A).

Inflammation is a hallmark of ALD. The hepatic pro-inflammatory cytokines, TNF-α, and IL-6 were markedly increased in binge alcohol exposure, an effect that was decreased by LP-cs pretreatment (Figure 2B, 2C). Similarly, the expression level of the hepatic p65 gene was enhanced by alcohol exposure, an effect that was inhibited by LP-cs pretreatment (Figure 2D). These findings suggest that alcohol-induced hepatic inflammation is reduced by LP-cs pretreatment.

### LP-cs ameliorates acute alcohol-induced liver apoptosis

**Figure 3:**
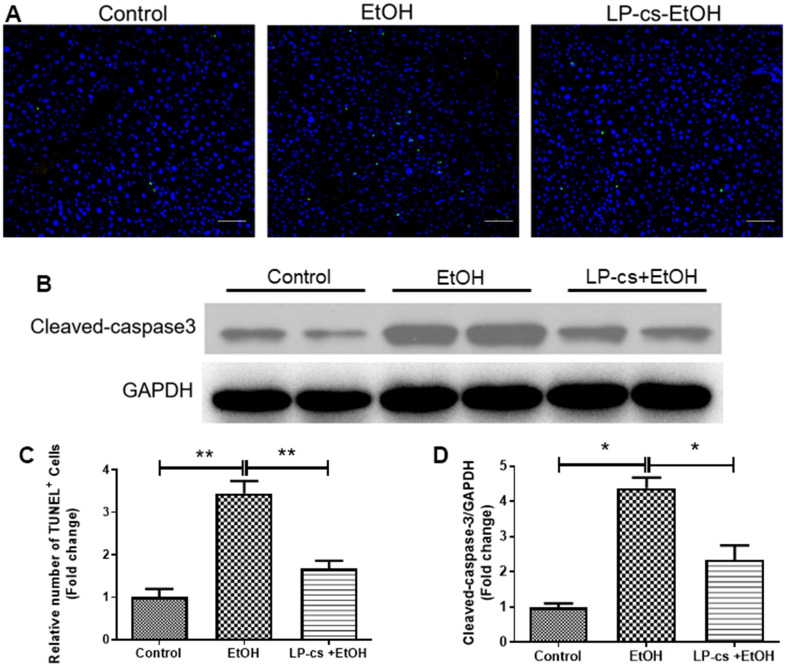
***Lactobacillus plantarum* ST-III culture supernatant (LP-cs) ameliorates acute alcohol-induced liver apoptosis.** (**A**) TUNEL-positive nuclear staining of liver from control, EtOH, LP-cs +EtOH mice (20 x: scale bars = 50 μm). (**B**) Protein expression of cleaved caspase-3 and GAPDH of liver from Control, EtOH, LP-cs +EtOH mice. (**C**) Quantification of relative number of TUNEL-positive cells. (**D**) Intensities of cleaved caspase-3 normalized to GAPDH. Data are expressed as mean ± SEM. *p < 0.05 and **p < 0.01.

Hepatic apoptosis was determined by TUNEL assay. The relative number of TUNEL^+^ cells was more significant in alcohol-treated mice than in controls, and this phenomenon was reversed by LP-cs pretreatment (Figure 3A, 3C). In addition, alcohol-treated cells exhibited a more significant number of apoptotic cells than control cells. The amount of cleaved caspase-3 was increased more than four-fold by alcohol treatment. Pretreatment with LP-cs markedly reversed these changes induced by alcohol exposure (Figure 3B, 3D). These findings suggest that LP-cs pretreatment prevents = alcohol-induced hepatocyte apoptosis.

### LP-cs ameliorates acute alcohol-induced ROS

**Figure 4:**
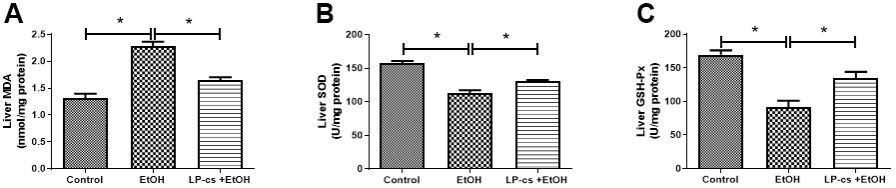
***Lactobacillus plantarum* ST-III culture supernatant (LP-cs) ameliorates acute alcohol-induced ROS.** (**A**) Liver malondialdehyde (MDA) levels. (**B**) Liver superoxide dismutase (SOD) levels. (**C**) Liver glutathione peroxidase (GSH-PX) levels. Data are expressed as mean ± SEM. *p < 0.05.

Alcohol exposure-induced oxidative stress is a significant contributor to ALD. We determined whether LP-cs would rescue the defective antioxidant system in alcohol-treated mice to ascertain the protective role in acute alcohol-induced hepatotoxicity. Compared with the control group, SOD and GSH-Px levels were significantly lower; however, MDA levels were markedly higher in the alcohol group. These effects were all reversed by LP-cs pre-treatment (Figure 4), suggesting that LP-cs inhibits acute alcohol-induced hepatic oxidative stress *in vivo*.

### LP-cs ameliorates acute alcohol-induced liver ER stress

**Figure 5:**
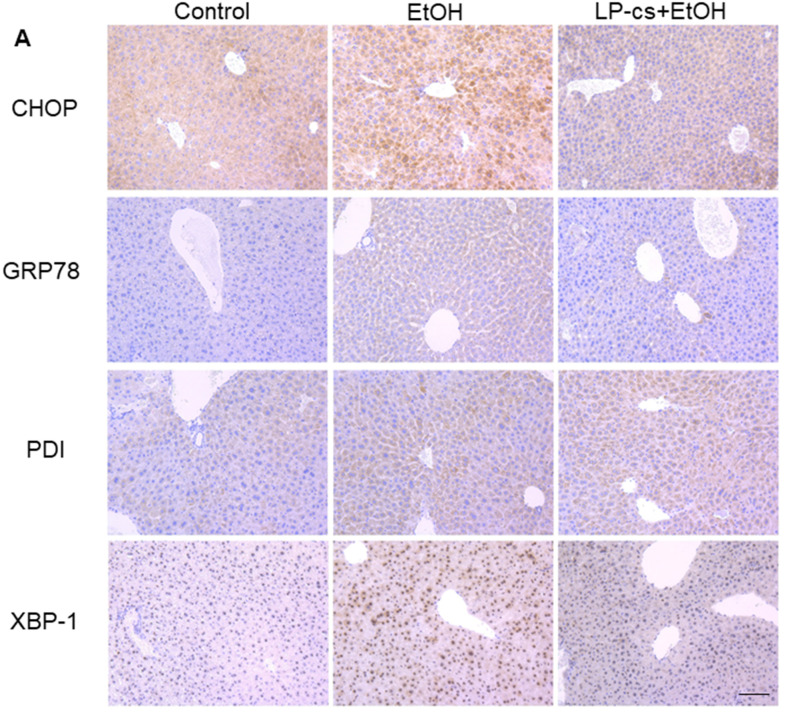
***Lactobacillus plantarum* ST-III culture supernatant (LP-cs) ameliorates acute alcohol-induced liver ER stress.** (**A**) Immunohistochemistry staining of C/Ebp-homologous protein (CHOP), glucose-regulated protein 78 (GRP78), protein disulfide isomerase (PDI), and X box-binding protein-1 (XBP-1) positive area of liver from control, EtOH, and LP-cs +EtOH mice (20 x: scale bars = 50 μm).

ER stress causes cellular toxicity through several signaling pathways, including apoptosis and autophagy. To assess the role of ER stress in alcohol-induced hepatotoxicity, immunohistochemistry staining was used to demonstrate the expression of the ER stress- associated proteins, including CHOP, GRP78, PDI, and XBP-1. These proteins were upregulated markedly by alcohol treatment at 6 h and were significantly downregulated by LP-cs pretreatment (Figure 5A), suggesting that pretreatment with LP-cs prevents acute alcohol-induced hepatotoxicity by inhibiting ER stress.

### LP-cs ameliorates acute alcohol-induced intestine injury

**Figure 6:**
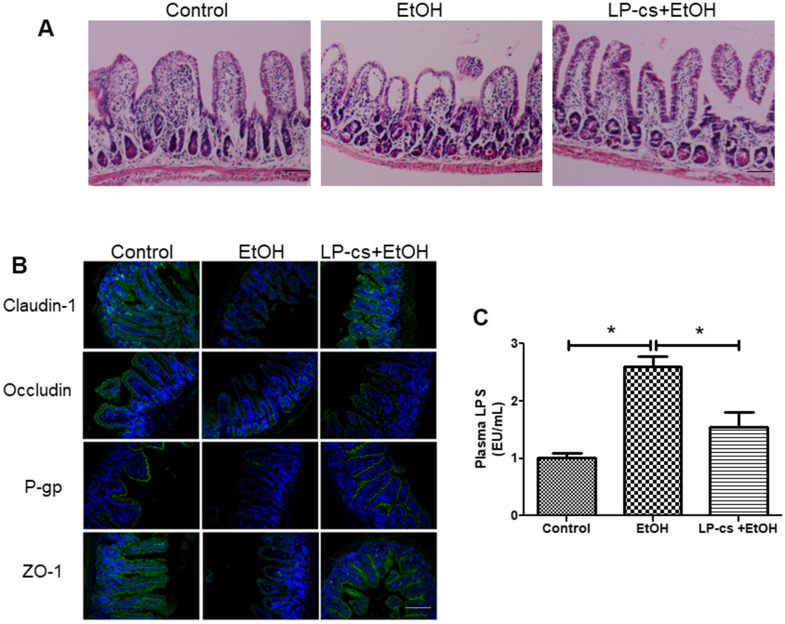
***Lactobacillus plantarum* ST-III culture supernatant (LP-cs) ameliorates acute alcohol-induced intestine injury.** (**A**) Hematoxylin and eosin (H&E) staining of intestine from control, EtOH, and LP-cs +EtOH mice (20 x: scale bars = 50 μm). (**B**) Immunofluorescence staining of claudin-1, occludin, P-gp, and zonula occludens-1 (ZO-1) positive area of intestine from Control, EtOH and LP-cs +EtOH mice (40 x: scale bars = 25 μm). (**C**) Plasma LPS levels. Data are expressed as mean ± SEM. *p < 0.05.

Histological staining of intestine sections revealed that alcohol triggered structural disruption of the central lacteal and reduced the number of small intestinal folds. These injuries were significantly reversed with LP-cs pretreatment (Figure 6A). These findings suggest that LP-cs pretreatment protects against alcohol-induced intestine injury.

### LP-cs protects intestinal barrier integrity

Tight junction proteins are critical to regulating intestinal barrier function. Alcohol exposure triggered a reduction in the distribution of claudin-1, occludin, and ZO-1 between adjacent epithelial cells in several parts of the ileal epithelium, and LP-cs pretreatment restored homogeneous distribution of tight junction proteins (Figure 6B). We also examined the expression of the intestinal P-gp protein, encoded by the multidrug resistance-1 gene. P-gp is widely expressed in intestinal epithelial cells and prevents harmful substances from damaging the intestine. The immunofluorescence results showed that P-gp expression was reduced by binge alcohol exposure, and LP-cs pretreatment reversed this reduction (Figure 6B). These findings suggest that LP-cs pretreatment protects against alcohol-induced intestinal barrier damage.

### Effects of LP-cs pretreatment on plasma endotoxemia

LPS is an endotoxin deriving from gram-negative bacteria and is a major pathogenic factor in ALD. Elevated plasma LPS levels resulted from intestine injury and defected in intestinal barrier integrity [29]. Alcohol exposure increases LPS production. LPS binds to Toll-like receptor 4 on the surface of hepatic Kupffer cells and activates the NF-κB-mediated TNF-α signaling pathway, causing hepatic steatosis and inflammation [30]. Plasma LPS levels were measured and analyzed 6 h after binge alcohol exposure to assess the effects of acute alcohol gavage and LP-cs pretreatment on plasma endotoxemia. Binge alcohol exposure markedly increased plasma LPS levels, and LP-cs pretreatment significantly reduced these elevations (Figure 6C).

## DISCUSSION

Acute alcohol-treated mice displayed significantly increased hepatic fat accumulation, inflammatory reactions, and apoptosis and also demonstrated severe damage to the intestinal mucosa with destruction of intestinal adhesion proteins. LP-cs pretreatment significantly alleviated alcohol-induced liver and intestine damage. LP-cs significantly reduced alcohol-induced oxidative and ER stress and protected hepatocytes from apoptosis. These findings suggest that the extracellular products of *L. plantarum* protect against alcohol-induced liver and intestinal injury.

Probiotics modulate intestine microbial homeostasis and manage diverse liver diseases, including nonalcoholic fatty liver disease, cirrhosis with hepatic encephalopathy, and ALD [31]. Pre-clinical studies reported that pre-treatment with probiotics can prevent ALI by repopulating gut flora and reducing alcohol-induced LPS and fat infusion [32]. However, many uncontrolled factors are involved in the substantial intake of probiotics, reflected in the biological characteristics instability, responsible for unpredictable side effects [33, 34]. Furthermore, studies have shown that microorganisms produce biologically active metabolites such as short-chain and conjugated fatty acids, extracellular polysaccharides, and neuroactive metabolites such as gamma-aminobutyric acid and serotonin, which can provide health benefits [35]. Therefore, this study used LP-cs to explore its effect on ALD to identify safer and more effective therapeutic drugs.

In the present study, alcohol exposure significantly increased hepatic lipid accumulation, liver injury, and hepatic apoptosis, while LP-cs pretreatment significantly prevented these changes. The protective role of LP-cs in fat accumulation might be associated with metabolism, including de novo lipogenesis and catabolism. During hepatic steatosis development, expression levels of hepatic SREBP-1c were upregulated in alcohol-treated mice, leading to increased gene expression involved in de novo lipogenesis (Figure 1C). Hepatic PGC-1α and PPAR-α gene expression were significantly decreased in alcohol-treated mice, causing fatty acid β-oxidation reduction. Pretreatment with LP-cs markedly reversed the changes in these genes induced by alcohol exposure (Figure 1D).

We also found that LP-cs significantly reduced alcohol-induced inflammation. Expression of TNF-α and IL-6, markers of acute inflammatory phase reaction, was significantly upregulated in response to binge alcohol exposure. LP-cs pretreatment prevented these increases (Figure 2B, 2C). Similarly, acute alcohol exposure increased the expression level of the hepatic p65 gene. The change was prevented by LP-cs pretreatment (Figure 2D). In addition, we found that LP-cs pretreatment played a role in hepatic defense to alcohol-induced hepatocyte apoptosis as measured by TUNEL assay and levels of cleaved caspase-3, an apoptosis-related protein (Figure 3). These findings suggest a role of LP-cs in anti-fat accumulation, inflammatory, and apoptosis activity in response to binge alcohol exposure.

Most experimental data indicated that oxidative stress plays a vital role in the onset and progression of alcohol-induced liver disease [14]. Some clinical studies found that probiotics ameliorate liver function by decreasing oxidative damage/stress [36]. In the present study, the levels of SOD and GSH-Px were significantly decreased, and MDA levels were markedly increased in the alcohol group, which were all reversed by LP-cs pre-treatment (Figure 4), suggesting that LP-cs significantly inhibited acute alcohol-induced hepatic oxidative stress.

There are conflicting reports regarding the role of ER stress in the etiology of ALD [37]. ALI has been studied using intragastric alcohol-fed mice, reproducing the pathology characteristics and progression of early ALI and demonstrating ER stress's involvement [38, 39]. The expression levels of ER stress-related genes, including GRP78, GRP94, CHOP/GADD153, and caspase 12, were upregulated, suggesting that ER stress response may lead to the pathologic features of ALD [40]. In the current study, immunohistochemistry revealed that ER stress-associated proteins GRP78, CHOP, XBP-1, and PDI were remarkably upregulated by alcohol treatment and were significantly downregulated by LP-cs pretreatment (Figure 5), suggesting that LP-cs pretreatment prevented acute alcohol-induced ER stress.

Alcohol leads to quantitative and qualitative gut flora alterations, mucosal damage, and gut permeability enhancement, resulting in the translocation of bacterial products and endotoxins into portal blood flow [7]. Bacterial products stimulate the production of pro-inflammatory mediators, including ROS, chemokines, cytokines, and leukotrienes, which cause inflammatory cell infiltration and liver injury, such as fibrosis [41]. In the present study, histological staining of intestine sections revealed that acute alcohol triggered structural disruption of the central lacteal and reduced the number of small intestinal folds. These injuries were significantly reversed with LP-cs pretreatment (Figure 6). Decreased expression levels of several TJ proteins (claudin-1, occludin, and ZO-1) in the intestine were reported in the experimental mouse model of ALD [42]. Oxidative stress induced by alcohol exposure significantly triggers the intestinal barrier's damage by reducing tight junctions [43]. The present study demonstrated that binge alcohol exposure downregulated intestinal tight junction protein levels, and LP-cs pretreatment reversed these changes (Figure 6). Additionally, P-gp (a 170-kDa transmembrane protein) is abundantly expressed on the apical surface of intestinal epithelial cells. Several lines suggest that P-gp protects the intestinal epithelia by mediating bacterial toxin efflux from the intestinal mucosa into the gut lumen. Dysregulated P-gp expression is associated with the pathogenesis of several gut disorders, such as inflammatory bowel disease, experimental animal models of colitis, ulcerative colitis, and Crohn’s disease. Upregulation of P-gp by two probiotic strains (*Lactobacillus acidophilus* and *rhamnosus)* has been demonstrated in a mouse model of DSS-induced colitis [42]. Our present findings suggest that binge alcohol exposure remarkably reduced P-gp expression levels. These changes were reversed by LP-cs pretreatment (Figure 6). Therefore, the protective effect against intestinal barrier dysfunction binge alcohol-induced might be achieved via a combinatorial regulation of intestinal mucin function. Furthermore, previous studies have identified that defects in intestinal barrier integrity can result in elevated plasma LPS levels in ALD models [44]. In the current study, acute alcohol exposure significantly increased plasma LPS levels, and LP-cs pretreatment significantly attenuated the rise in LPS levels (Figure 6).

LP-cs contains many short-chain fatty acids (SCFAs), proteins, polypeptides, and other potential cellular components or organic metabolites [45]. Acetic acid regulates intestinal pH, maintaining the stability of the intestinal microenvironment, nourishing beneficial microorganisms, and preventing the invasion of opportunistic pathogens. SCFAs play an essential role in maintaining normal intestinal function and the morphology and function of colonic cells, the intestinal morphology [45]. SCFAs are beneficial carbohydrates the intestinal microbiota produces [46]. Acetic acid benefits butyric acid-producing bacteria in the intestine and the diversity of beneficial flora [45]. *Firmicutes* use acetic acid to produce butyric acid. Butyric acid is the primary energy source of colon cells (>90%). Butyric acid maintains the integrity of the intestinal wall and prevents pathogenic bacteria, toxins, and other harmful substances from entering the circulation [47].

In summary, we found that binge alcohol exposure damaged mucus protective protein regulation involved in the expression of TJ proteins, LPS, and eventually ALI. LP-cs helped maintain liver and intestinal histomorphology, antioxidation, anti-ER stress, and intestinal microbial homeostasis on alcoholic stress. We will test other components to demonstrate LP-cs' effect on alcohol-induced liver injury in future. Further characterization of the LP-cs active components and different ALD models will improve our understanding of the protective effect of probiotics on ALD and advance the development of novel therapeutic strategies for ALD.
